# Comparison of Stromal Vascular Fraction and Adipose-Derived Stem Cells for Protection of Renal Function in a Rodent Model of Ischemic Acute Kidney Injury

**DOI:** 10.1155/2022/1379680

**Published:** 2022-05-06

**Authors:** Joomin Aum, Myoung Jin Jang, Yu Seon Kim, Bo Hyun Kim, Dong Hyeon An, Jae Hyeon Han, Nayoung Suh, Choung-Soo Kim, Dalsan You

**Affiliations:** ^1^Department of Urology, Asan Medical Institute of Convergence Science and Technology, Asan Medical Center, University of Ulsan College of Medicine, Seoul, Republic of Korea; ^2^Asan Institute for Life Sciences, Asan Medical Center, Seoul, Republic of Korea; ^3^Department of Urology, Asan Medical Center, University of Ulsan College of Medicine, Seoul, Republic of Korea; ^4^Department of Urology, Korea University Ansan Hospital, Korea University College of Medicine, Ansan, Republic of Korea; ^5^Department of Pharmaceutical Engineering, College of Medical Sciences and Department of Medical Sciences, General Graduate School, Soon Chun Hyang University, Asan, Republic of Korea

## Abstract

**Aims:**

Few studies have compared the use of different cell types derived from adipose tissue or the optimal route for efficient and safe cell delivery in ischemic acute kidney injury (AKI). We compared the abilities of stromal vascular fraction (SVF) and adipose-derived stem cells (ADSC), injected via three different routes, to protect renal function in a rodent model of ischemic AKI.

**Methods:**

Ninety male Sprague-Dawley rats were randomly divided into 9 groups: sham, nephrectomy control, AKI control, transaortic renal arterial SVF injection, renal parenchymal SVF injection, tail venous SVF injection, transaortic renal arterial ADSC injection, renal parenchymal ADSC injection, and tail venous ADSC injection groups. Their renal function was assessed 4 days before and 1, 2, 3, 4, 7, and 14 days after surgical procedures to induce ischemic AKI. The histomorphometric studies were performed 14 days after surgical procedures.

**Results:**

Renal parenchymal injection of SVF notably reduced the level of serum blood urea nitrogen and creatinine elevation compared to the AKI control group. Renal parenchymal injection of SVF notably reduced the level of creatinine clearance decrease. In addition, collagen content was lower in the renal parenchymal SVF injection group, and fibrosis was reduced. Apoptosis was reduced in the renal parenchymal SVF injection group, and proliferation was increased. The expression levels of antioxidative markers such as glutathione reductase and peroxidase were higher in the renal parenchymal SVF injection group.

**Conclusions:**

Our findings suggest that renal function is protected from ischemic AKI through renal parenchymal injection of SVF, which has enhanced antifibrotic, antiapoptotic, and antioxidative effects.

## 1. Introduction

Acute kidney injury (AKI), also known as acute renal failure, is characterized by abrupt deterioration in renal function. AKI causes a build-up of nitrogenous waste products in blood and makes it hard for the kidneys to maintain fluid and electrolyte homeostasis [1]. Renal ischemia and subsequent reperfusion injury is a common etiology of AKI in hospitals [1, 2]. Ischemic AKI is an inevitable consequence of kidney transplantation from deceased donors, on-pump cardiac surgery, and nephron-sparing surgery. Unfortunately, current treatments to reduce the extent of ischemic AKI during these surgeries are primarily supportive.

Recently, cell-based therapies have been applied with some promising results in AKI [3–8]. Both cultured and noncultured cells have been demonstrated to improve tissue regeneration and functional recovery in AKI [9–13]. We previously found that renal arterial injection of hypoxic preconditioned bone marrow-derived mesenchymal stem cells (MSC) or adipose-derived stromal vascular fraction (SVF) rescues renal function in a rodent model of ischemic AKI [14, 15]. In spite of several preclinical studies demonstrating the efficiency of cell-based therapy in treating kidney diseases, clinical trials are only in the very early stages and have been associated with some problems such as determining the optimal cell sources and delivery method [16].

To avoid various risks regarding transplanting cultured stem cells into humans [17], noncultured SVF has been proposed as a simpler and safer method using adipose-derived stem and progenitor cells. Recent clinical trials have focused on the use of noncultured SVF in the treatment of various kidney diseases [18–20]. However, there has been only limited research comparing various adipose-derived cells used to treat ischemic AKI [21]. In addition, the optimal route for efficient and safe cell delivery is not yet known [14]. Therefore, we compared the efficacy and safety of SVF and adipose-derived stem cells (ADSC), injected via three different routes, to protect renal function in a rodent model of ischemic AKI.

## 2. Materials and Methods

### 2.1. Animal Care and Study Design

The protocol for animal experimentation was reviewed and approved by the Institutional Animal Care and Use Committee of Asan Institute for Life Sciences. Ninety 11-week-old male Sprague-Dawley rats (ORIENT BIO Inc., Seongnam, Korea, http://www.orient.co.kr) were used. After 1-week acclimatization period, the rats were randomly divided into 9 groups: sham, nephrectomy control (NX control), AKI control, transaortic renal arterial SVF injection (SVF-AA), renal parenchymal SVF injection (SVF-RP), tail venous SVF injection (SVF-TV), transaortic renal arterial ADSC injection (ADSC-AA), renal parenchymal ADSC injection (ADSC-RP), and tail venous ADSC injection (ADSC-TV) groups. Rats in the ADSC injection groups underwent bilateral paratesticular fat excision via scrotal incision to culture ADSC. At 14 weeks, rats in the SVF injection groups underwent bilateral paratesticular fat excision to isolate SVF. Surgical procedures were performed; then, SVF and ADSC were injected into different three routes in the SVF and ADSC injection groups, respectively. The renal function was assessed 4 days before and 1, 2, 3, 4, 7, and 14 days after surgical procedures. At 16 weeks old, all rats were sacrificed, and their left kidney was harvested for histological examination.

### 2.2. Isolation of SVF and Culture of ADSC and Characterization

SVF consists of a heterogeneous population of cells including ADSC, endothelial cells, and endothelial precursor cells, whereas ADSC are a homogeneous population of one type of cells [22]. Adipose tissue was harvested from each rat's own paratesticular fat at the time of randomization (ADSC injection groups) or shortly before surgical procedures (SVF injection groups). SVF isolation and ADSC culture were performed as described previously in detail [23]. Flow cytometry to characterize SVF and ADSC was performed with a FACScan argon laser cytometer (BD Biosciences, San Jose, CA, http://www.bdbiosciences.com) as described previously in detail [23]. Differentiation of ADSC was confirmed as described previously in detail [23].

### 2.3. Labeling and Tracking

SVF and ADSC were labeled with the fluorescent dye CM-DiI (Thermo Fisher Scientific Inc., Waltham, MA, https://corporate.thermofisher.com). Cells were resuspended at a density of 1 × 10^6^ cells/mL in serum-free DMEM medium. The cell suspension was then mixed with 5 *μ*L of CM-DiI cell labeling solutions per milliliter and incubated at 37°C for 30 min. An equal volume of serum-containing DMEM medium was added to stop the staining reaction. The labeled suspension was centrifuged, and the supernatant was removed. The cells were resuspended in serum-free DMEM medium and injected into the rats through each route. Cross section of left kidney was observed using the fluorescent microscope.

### 2.4. Surgical Procedures and Cell Transplantation

After anesthesia, surgical procedures and cell transplantation were performed as described previously in detail [14]. Laparotomy and right nephrectomy were performed in the sham group and NX control group, respectively. With right nephrectomy, ischemic AKI of the left kidney was induced in the other seven groups. Ischemic AKI was induced as described previously in detail [14, 15]. In the renal arterial injection groups, 100 *μ*L of a cell suspension in PBS (1 × 10^6^ cells) was injected into the aorta after clamping the aorta above and below the left renal artery using bulldog clamps (Fine Science Tools, Heidelberg, Germany, https://www.finescience.com) shortly before the induction of ischemic AKI. Then, the left renal artery was clamped with a bulldog clamp for 40 min. A 33-gauge Hamilton syringe (Hamilton Company, Zurich, Switzerland, https://www.hamiltoncompany. com) was used to minimize the damage to the aorta. The injection site was closed using TachoSil® Sealant matrix (Takeda Austria GmbH, Linz, Austria, https://www.takeda.com), and the clamps were removed to restore aortic blood flow after 10 min. In the renal parenchymal injection groups, a 33-gauge Hamilton syringe containing the same volume of cell suspension was penetrated the kidney longitudinally. While slowly pulling out the needle, the cells were gradually released from the syringe into the kidney. In the tail venous injection groups, the same volume of cell suspension was injected via the tail vein using a 26-gauge Kovax-syringe (Koreavaccine, Ansan, Korea, http://www.koreavaccine.com).

### 2.5. Observation of Mortality and Measurements of Body Weight and Food Consumption

All rats were observed daily, and mortality was recorded. Their body weight was measured 4 days before and 1, 2, 3, 4, 7, and 14 days after surgical procedures. Their food consumption was measured weekly prior to surgical procedures and during the observational period. The amount of food consumed was estimated by subtracting the amount of leftover food from the amount of presented food.

### 2.6. Determination of Renal Function

Serum blood urea nitrogen (BUN) and serum and urine creatinine levels were measured 4 days before and 1, 2, 3, 4, 7, and 14 days after surgical procedures. Urine was collected from the rats in a metabolic cage to estimate the 24 h urine volume. The creatinine clearance (CrCl) was computed with the following formula and normalized to body weight [24]: CrCl (mL/min/100 g) = urine creatinine (mg/dL) × 24 h urine volume (mL) × 100/serum creatinine (mg/dL) × 1440 × body weight (g).

### 2.7. Kidney Harvest

The weight of left kidney was measured immediately after harvest. Half of each kidney was cryopreserved in liquid nitrogen, and the remaining half was fixed in 4% paraformaldehyde and embedded in paraffin. The renal index was computed with the following formula [25]: renal index (mg/g) = kidney weight (mg)/body weight measured 14 days after surgical procedures (g).

### 2.8. Hematoxylin and Eosin Staining and Histopathological Score

Kidney tissues were sectioned at 4 *μ*m intervals, and hematoxylin and eosin staining was performed by standard methods. Histopathological score was estimated based on methods used in previous studies in a blinded manner [26]. Histopathological score was quantitated by calculation of the degree of cast formation, interstitial inflammation, tubular dilatation, and glomerulus degeneration as follows: 0 (none), 1 (≤24%), 2 (25-49%), 3 (50-74%), and 4 (≥75%).

### 2.9. Sirius Red Staining and Masson's Trichrome Staining

Sirius red staining was performed as described previously in detail [14]. After drying for 1 h in a 60°C oven, the slides were sequentially washed with xylene I, xylene II, and xylene III and then rehydrated through graded ethanol solutions, 1 min each, and washed in distilled water 3 times. They were then stained with Harris hematoxylin solution (Sigma-Aldrich, St. Louis, MO, https://www.sigmaaldrich.com) for 5 min. Then, the slides were rinsed three times in distilled water, and the area around the tissue was marked with a PAP pen. Pico Sirius Red reagent 60 *μ*L was dropped on the slides and incubated for 20 min. After dipping twice in an acid solution, the slides were washed three times with distilled water, then dehydrated through graded ethanol. The sections were cleared by incubation in xylenes I, II, and III for 10 min each and mounted using permanent mounting media. The whole kidney slides of each rat were digitized with the Panoramic SCAN digital slide scanner (3DHISTECH Ltd., Budapest, Hungary, https://www.3dhistech.com), and the histological images were subsequently analyzed by the panoramic case viewer software (3DHISTECH Ltd.) at ×20 magnification. For image analysis, four randomly selected cortex and medulla fields of kidney in all rats were photographed via CaseViewer of 3DHISTECH. All images were analyzed using Adobe Photoshop CS2 to quantify signals. The results of Sirius red staining were presented as the percentage of collagen positive areas to the total area in cortex or medulla of each field.

Masson's trichrome staining was performed as described previously in detail [23]. After staining, it was analyzed in the same way as mentioned for Sirius red staining. The results of Masson's trichrome staining were presented as the percentage of fibrotic area (blue-colored) in cortex or medulla of each field.

### 2.10. Western Blot Analysis

The sectioned kidney tissues were incubated with lysis buffer (iNtRON Biotechnology Inc., Seongnam, Korea, http://www.intronbio.com) on ice for 20 min. Collected lysates were boiled for 5 min and separated on a 9% gradient acrylamide gel and then transferred electrophoretically to polyvinyl difluoride membranes (MilliporeSigma, Burlington, MA, https://www.sigmaaldrich.com). Nonspecific binding of antibody was blocked with 5% bovine serum albumin. Immunodetection was performed with to an anti-*α* smooth muscle actin (*α*-SMA) primary antibody (1 : 2,000; Abcam, Cambridge, U.K., http://www.abcam.com) followed by incubation with the peroxidase-conjugated secondary antibody (1 : 5,000; Enzo Life Sciences, Farmingdale, NY, https://www.enzolifesciences.com), and the blots were developed using SuperSignal West Pico Chemiluminescent Substrate (Thermo Fisher Scientific Inc.). Primary and secondary antibodies were diluted in 5% skim milk (bioWORLD, Dublin, OH, http://www.bio-world.com) in PBS. All blots were normalized against *α*-tubulin to control for protein loading. The immunoblot bands were detected using Immobilon™ Western HRP substrate peroxide Solution (Thermo Fisher Scientific Inc.).

### 2.11. TUNEL Assays

Terminal deoxynucleotidyl transferase- (TdT-) mediated dUTP nick end labeling (TUNEL) for the detection of apoptotic cells was performed using an in situ cell death detection kit according to the manufacturer's protocol (Roche Molecular Biochemicals, Mannheim, Germany, https://lifehttp://science.roche.com/shop/home). Under light microscopy, TUNEL-stained sections were observed, and the positive cells were counted. TUNEL staining was performed as described previously in detail [15].

### 2.12. Immunohistochemistry

Histological sections were labelled with anti-Ki67 (1 : 200, Abcam), antiglutathione reductase (GR; 1: 2000, Abcam), or antiglutathione peroxidase (GPx; 1 : 2000, Abcam) antibody as described previously in detail [14]. After staining, it was analyzed in the same way as mentioned for Sirius red staining. The results of immunohistochemistry were presented as the percentage of antibody positive areas to the total area in cortex or medulla of each field.

### 2.13. Statistical Analysis

Continuous variables are expressed as mean ± standard error. Multiple groups were compared using one-way analysis of variance followed by Tukey's honest significant difference test for post hoc comparisons. Nonparametric analyses using Kruskal-Wallis test followed by Mann–Whitney *U* test for post hoc comparisons were used for statistical analysis of renal index, histopathological score, and Western blot analysis. All statistical tests were two-sided, and statistical significance was defined as *p* < 0.05, *p* < 0.01, or *p* < 0.001; *p* ≥ 0.05 was not considered significant. The data were analyzed using SPSS Statistics, version 21 (IBM Corp., Armonk, NY, http://www.ibm.com).

## 3. Results

### 3.1. Characterization of SVF and ADSC

**Figure 1:**
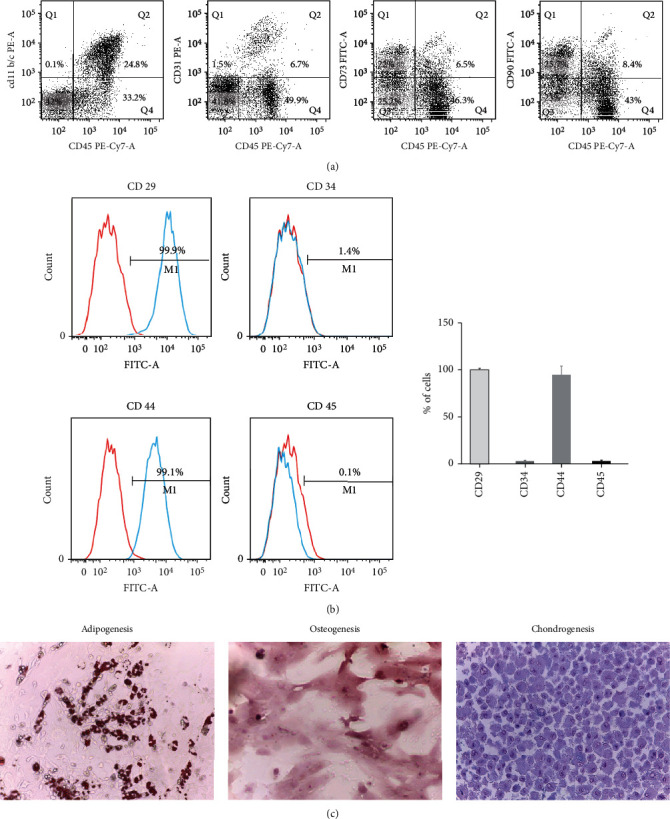
Characterization of SVF and ADSC. (a) Representative flow cytometry histograms of SVF. 1 time using SVF collected from 3 rats, 3 rats per each SVF group. (b) Flow cytometry of ADSC. Results were expressed as mean ± standard error of all independent experiments. 3 times per rat, 3 rats per each ADSC group. (c) Appearance of ADSC after 3 weeks of induction of adipogenic, osteogenic, and chondrogenic differentiation. Magnification, ×100 in adipogenesis and osteogenesis and ×400 in chondrogenesis. 3 times, 3 rats per each ADSC group.

Flow cytometric analysis indicated that SVF consisted of more than half of CD45+ cells (blood-derived cells), 1.5% of CD45-/CD31+ (endothelial cells), and 42% of CD45-/CD31- cells. CD73 and CD90 were expressed by almost half of CD45- cells (stromal cells). Flow cytometric analysis indicated that 98.8% of ADSC expressed CD29, 1.1% expressed CD34, 94.0% expressed CD44, and 1.1% expressed CD45. Their multilineage differentiation ability was confirmed by observation of adipogenic, osteogenic, and chondrogenic differentiation after 3 weeks incubation in the appropriate induction media (Figure 1).

### 3.2. Mortality, Body Weight, and Food Consumption

Two rats in the SVF-AA group died from AKI on days 4 and 7 after ischemic AKI, respectively. One rat in the ADSC-AA group experienced intraoperative death due to anesthesia. Another rat in the ADSC-AA group died from hemorrhage day 1 after ischemic AKI. There was no significant difference in body weight or food consumption among the groups.

### 3.3. Renal Parenchymal Injection of SVF Ameliorate Renal Function

**Figure 2:**
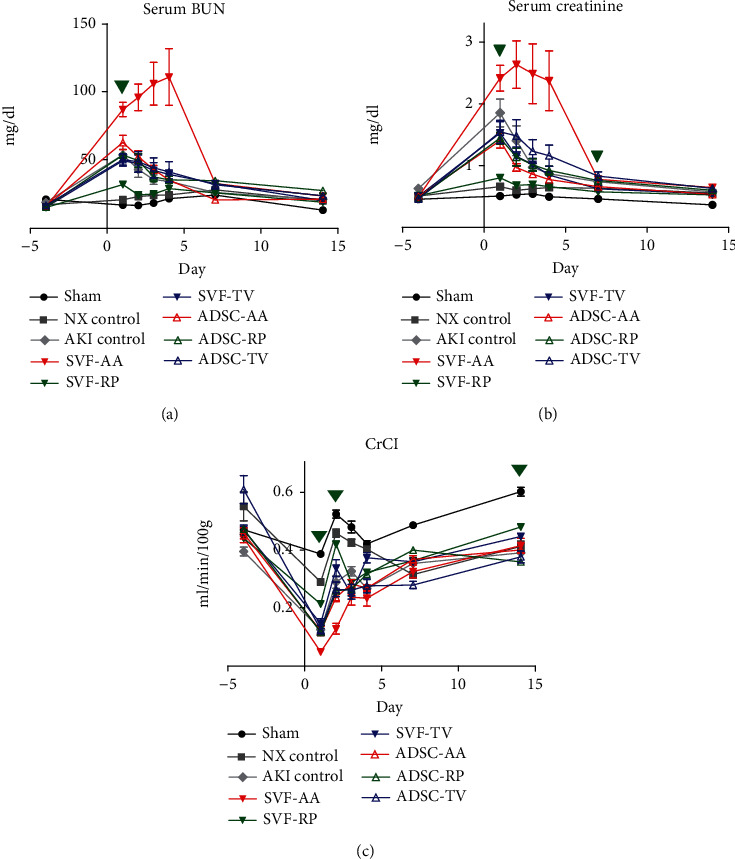
Effects of SVF and ADSC on renal function. Graphs showing changes in (a) serum BUN, (b) serum creatinine, and (c) CrCl of each group during the experiment. Green- and blue-colored inverted triangles indicate statistically significant differences in the SVF-RP and SVF-TV groups compared to the AKI control group, respectively. 3 times per rat; 10 rats per group, but only until the time of survival of rats in the SVF-AA and ADSC-AA groups.

Renal parenchymal injection of SVF notably reduced the level of serum BUN elevation compared to the AKI control group 1 day after ischemic AKI (31.85 mg/dL vs. 53.53 mg/dL, *p* < 0.001) (Figure 2(a)). Renal parenchymal injection of SVF notably reduced the level of serum creatinine elevation compared to the AKI control group on days 1 and 7 after ischemic AKI (0.82 mg/dL vs. 1.87 mg/dL, *p* < 0.001; 0.59 mg/dL vs. 0.75 mg/dL, *p* < 0.05) (Figure 2(b)). Renal parenchymal injection of SVF notably reduced the level of CrCl decrease compared to the AKI control group on days 1, 2, and 14 after ischemic AKI (0.22 mL/min/100 g vs. 0.13 mL/min/100 g, *p* < 0.001; 0.42 mL/min/100 g vs. 0.29 mL/min/100 g, *p* < 0.001; 0.48 mL/min/100 g vs. 0.39 mL/min/100 g, *p* < 0.01). Tail venous injection of SVF notably reduced the level of CrCl decrease compared to the AKI control group 4 days after ischemic AKI (0.38 mL/min/100 g vs. 0.27 mL/min/100 g, *p* < 0.01) (Figure 2(c)).

Renal index was notably higher in the AKI control group than in both the sham and NX control groups. There was no significant difference in renal index between the AKI control and all injection groups (Figure S1).

### 3.4. Histopathological Score and Fibrosis of Kidney Tissue

**Figure 3:**
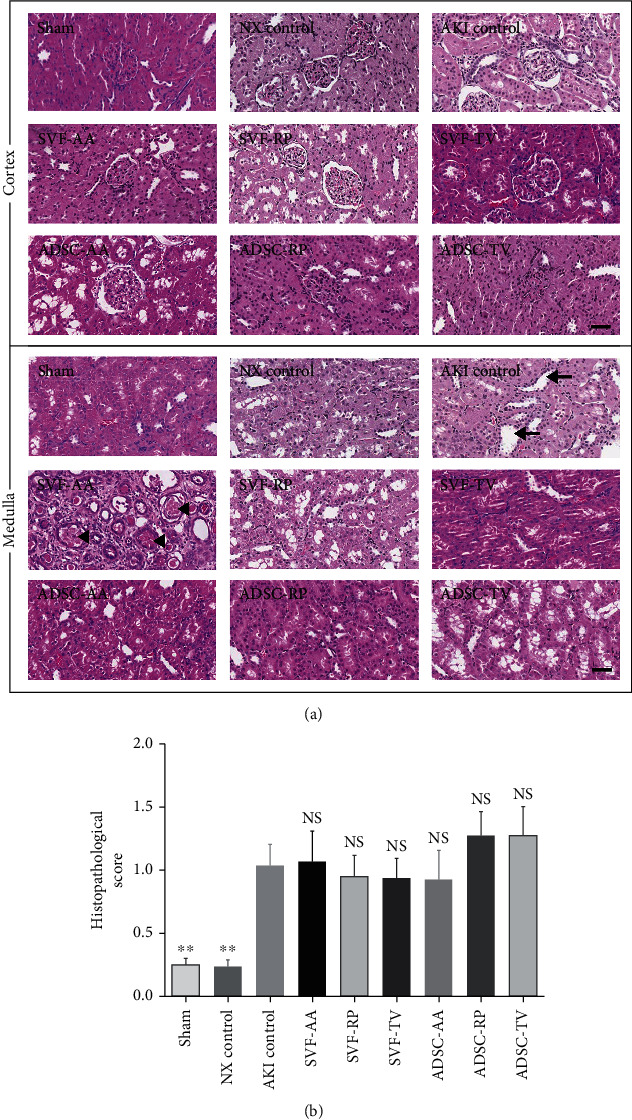
Histopathological score of kidney tissue. (a) Representative photomicrographs of cortex and medulla. Arrows and arrowheads indicate tubular dilatation and cast formation, respectively. (b) Histopathological score in random nonoverlapping fields. Magnification ×40. Scale bar 50 *μ*m. NS, *p* > 0.05; ^∗∗^*p* < 0.01 compared to the AKI control group. 10 fields per rat; 10 rats per group, but 8 rats in the SVF-AA and ADSC-AA groups.

Histopathological score was notably higher in the AKI control group than in both the sham and NX control groups. There was no significant difference in histopathological score between the AKI control and all injection groups (Figure 3).

**Figure 4:**
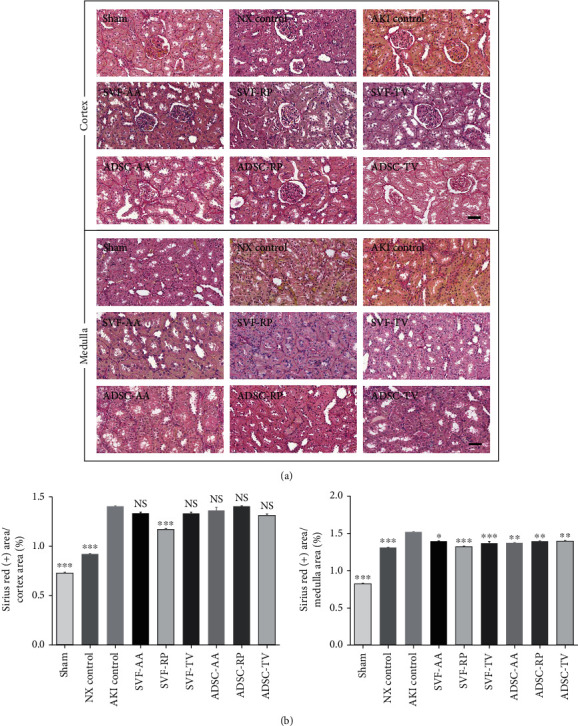
Sirius red staining of kidney tissue. (a) Representative photomicrographs of cortex and medulla. (b) Ratio of area stained positive for Sirius red to random cortex area (left) and medulla area (right). Magnification ×40. Scale bar 50 *μ*m. NS, *p* > 0.05; ^∗^*p* < 0.05; ^∗∗^*p* < 0.01; ^∗∗∗^*p* < 0.001 compared to the AKI control group. 4 fields per rat; 10 rats per group, but 8 rats in the SVF-AA and ADSC-AA groups.

**Figure 5:**
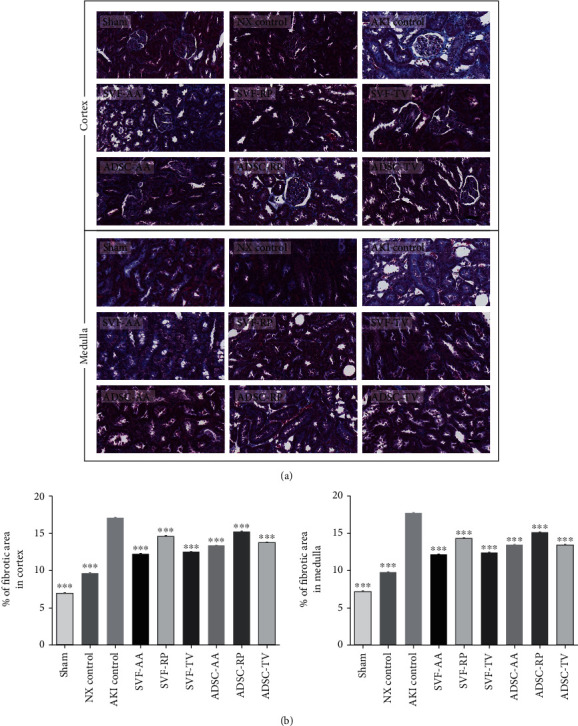
Masson's trichrome staining of kidney tissue. (a) Representative photomicrographs of cortex and medulla. (b) Percentage of fibrotic area in random cortex area (left) and medulla area (right). Magnification ×40. Scale bar 50 *μ*m. ^∗∗∗^*p* < 0.001 compared to the AKI control group. 4 fields per rat; 10 rats per group, but 8 rats in the SVF-AA and ADSC-AA groups.

**Figure 6:**
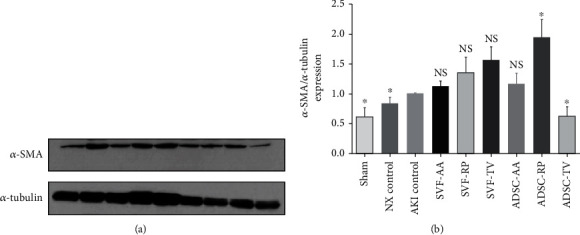
(a) Representative immunoblot bands of *α*-SMA expression in kidney tissue. (b) The graph showing protein levels as relative expression compared to the AKI control group. Relative quantification of protein levels was normalized for *α*-tubulin. NS, *p* > 0.05; ^∗^*p* < 0.05 compared to the AKI control group. 3 timed per rat; 3 rats per group.

Sirius red staining revealed that the collagen content was notably higher in the AKI control group than in both the sham and NX control groups in both cortex and medulla. Collagen content was notably lower in the SVF-RP group than in the AKI control group in both cortex and medulla (Figure 4). Masson's trichrome staining revealed a significant increase of fibrosis in the AKI control group compared to the sham and NX control groups in both cortex and medulla. Fibrosis was significantly reduced in all injection groups compared to the AKI control group in both cortex and medulla (Figure 5). On Western blot analysis, the expression of *α*-SMA was significantly increased in the AKI control group compared to the sham and NX control groups. After cell-based therapy, the expression of *α*-SMA was significantly increased in the ADSC-RP group compared to the AKI control group, whereas the ADSC-TV group showed significant decrease (Figure 6).

### 3.5. Apoptosis and Proliferation Markers of Kidney Tissue

**Figure 7:**
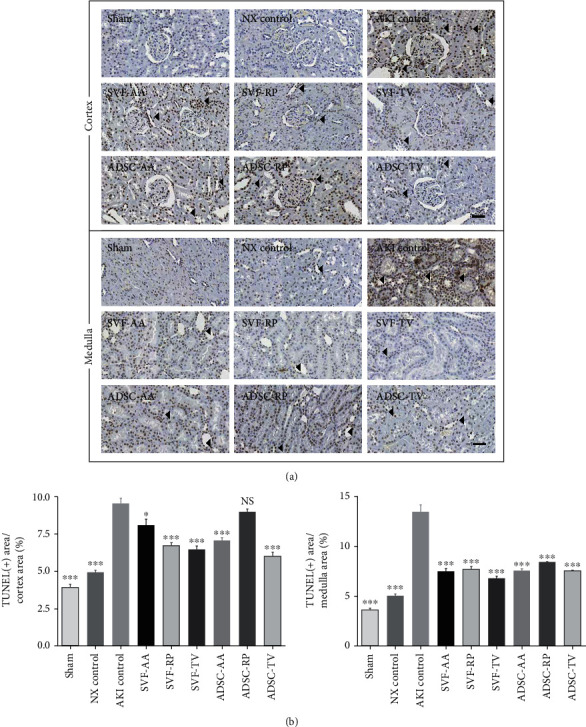
Apoptosis marker of kidney tissue. (a) Representative photomicrographs of cortex and medulla. Arrowheads indicate nuclei stained positive for TUNEL. (b) Ratio of area stained positive for TUNEL to random cortex area (left) medulla area (right). Representative photomicrographs of (right). Magnification ×40. Scale bar 50 *μ*m. NS, *p* > 0.05; ^∗^*p* < 0.05; ^∗∗∗^*p* < 0.001 compared to the AKI control group. 4 fields per rat; 10 rats per group, but 8 rats in the SVF-AA and ADSC-AA groups.

TUNEL assay revealed a significant increase of apoptosis in the AKI control group compared to the sham and NX control groups in both cortex and medulla. Apoptosis was significantly reduced in the SVF-AA, SVF-RP, SVF-TV, ADSC-AA, and ADSC-TV groups compared to the AKI control group in cortex and in all injection groups in medulla, respectively (Figure 7).

**Figure 8:**
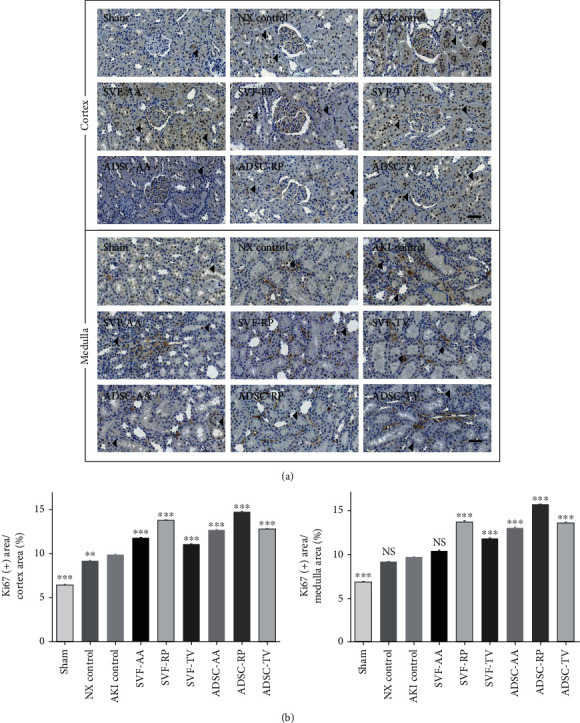
Proliferation marker of kidney tissue. (a) Representative photomicrographs of cortex and medulla. Arrowheads indicate nuclei stained positive for Ki67. (b) Ratio of area stained positive for Ki67 to random cortex area (left) and medulla area (right). Magnification ×40. Scale bar 50 *μ*m. NS, *p* > 0.05; ^∗∗^*p* < 0.01; ^∗∗∗^*p* < 0.001 compared to the AKI control group. 4 fields per rat; 10 rats per group, but 8 rats in the SVF-AA and ADSC-AA groups.

Ki67 staining revealed a significant increase of proliferation in the AKI control group compared to the sham group in both cortex and medulla. Proliferation was significantly increased in all injection groups compared to the AKI control group in cortex and in the SVF-RP, SVF-TV, ADSC-AA, ADSC-RP, and ADSC-TV groups in medulla, respectively (Figure 8).

### 3.6. Antioxidative Markers of Kidney Tissue

**Figure 9:**
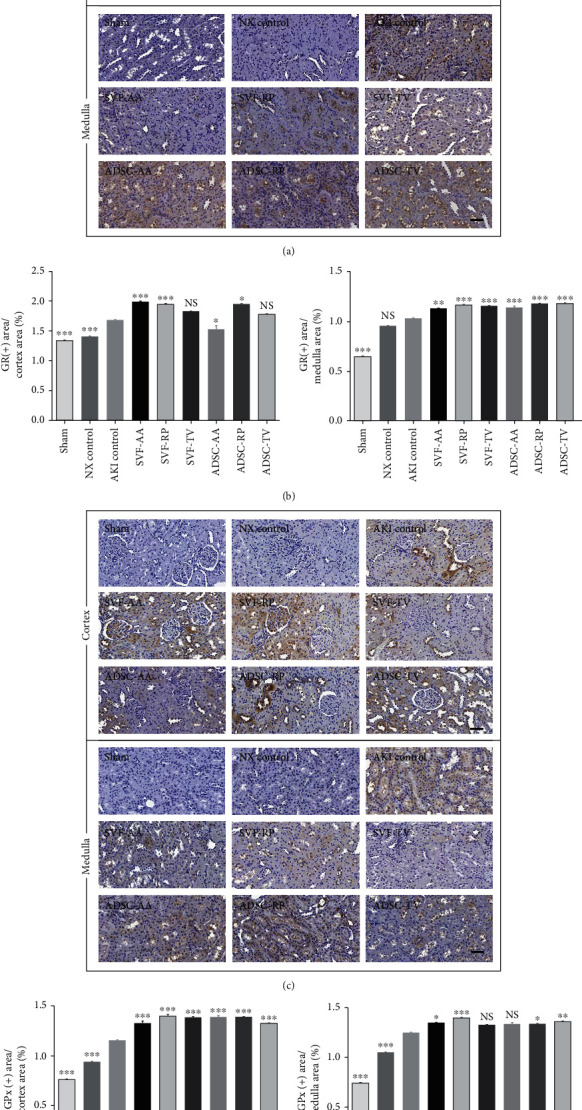
Antioxidation markers of kidney tissue. (a) Representative photomicrographs of cortex and medulla in GR. (b) Ratio of area stained positive for GR to random cortex area (left) and medulla area (right). (c) Representative photomicrographs of cortex and medulla in GPx. (d) Ratio of area stained positive for GPx to random cortex area (left) and medulla area (right). Magnification ×40. Scale bar 50 *μ*m. NS, *p* > 0.05; ^∗^*p* < 0.05; ^∗∗^*p* < 0.01; ^∗∗∗^*p* < 0.001 compared to the AKI control group. 4 fields per rat; 10 rats per group, but 8 rats in the SVF-AA and ADSC-AA groups.

The proportion of GR-positive area was notably higher in the AKI control group than in the sham group in both cortex and medulla. It was notably higher in the SVF-AA, SVF-RP, and ADSC-RP groups than in the AKI control group in cortex and in all injection groups in medulla, respectively (Figures 9(a) and 9(b)).

The proportion of GPx-positive area was notably higher in the AKI control group than in both the sham and NX control groups in both cortex and medulla. It was notably higher in all injection groups than in the AKI control group in cortex and in the SVF-AA, SVF-RP, ADSC-RP, and ADSC-TV groups in medulla, respectively (Figures 9(c) and 9(d)).

### 3.7. SVF and ADSC Cells Do Not Engraft in Kidneys

Two weeks after injection in all injection groups, neither CM-DiI-labeled SVF nor ADSC were found in the kidney tissue (Figure S2).

## 4. Discussion

Many preclinical studies have investigated the effects of cultured and uncultured cells isolated from various sources to treat AKI [27, 28]. Adipose tissue is a superior source of cultured and noncultured cells for tissue repair and regeneration for a number of reasons [29]. However, cultured cells have several limitations, including the cost and time of culture, risk of contamination, changes in cell characteristics during culturing procedures, and the risk of developing tumors [17]. Because of these limitations, preclinical studies using noncultured SVF to treat renal diseases have become increasingly abundant [9, 10, 15]. Various angiogenic factors secreted by SVF, such as hepatocyte growth factor (HGF), vascular endothelial growth factor-A, and stromal cell-derived factor-1*α*, play an important role in recuperating renal function [21]. There are, however, currently no clinical trials in progress to investigate the efficacy and safety of the use of SVF for treating ischemic AKI. In addition, the optimal route for efficient and safe cell delivery for treating ischemic AKI is currently unclear [14]. Depending on the route of delivery, cells may localize in the target organ or become trapped in nontarget organs, including the lungs, causing adverse effects and diminished therapeutic efficacy [30, 31]. This led us to perform this study. We directly compared SVF with ADSC, injected via three different routes, for their effectiveness in treating ischemic AKI.

We found that renal parenchymal injection of SVF most effectively restored renal function in a rodent model of ischemic AKI. Moreover, the SVF-RP group was significantly superior to the AKI control group in terms of all histomorphometric measures except for the histopathological score. Additionally, a significant increase in CrCl 4 days after ischemic AKI was detected in the SVF-TV group. However, previous studies investigating ischemic AKI have shown contradictory results in terms of cell sources or delivery routes.

Zhou et al. [21] compared renal parenchymal injection of xenogeneic SVF and ADSC *in vivo* and showed equal effectiveness in attenuating ischemic AKI. Unlike their study, our study demonstrated that only SVF showed significant attenuation of ischemic AKI. There are several explanations for this difference between the two studies. First, Zhou et al. [21] reported that SVF showed stronger enhancement of renal tubular cell proliferation than ADSC *in vitro* with more secretion of HGF by SVF. HGF can enhance the proliferation activity of renal tubular cells. Another explanation is that they used xenogeneic SVF and ADSC, whereas we used autologous SVF and ADSC. ADSC have no immune response [32, 33], but SVF consists of heterogeneous cell populations, including blood-derived cells, endothelial cells, and adipose-derived stromal cells that can cause an immune response after xenotransplantation. Therefore, xenogeneic SVF are less effective than autologous SVF. The ineffectiveness of ADSC in our study might, at least in part, be attributable to the lower number of cells used than in their study (1 × 10^6^ vs. 2 × 10^6^ cells).

In terms of delivery routes, Yasuda et al. [10] reported that subcapsular injection of autologous SVF limits renal injury via promoting renal capillary regeneration and proliferating renal tubular cells. Our previous study evaluated the optimal route for autologous SVF delivery and found modest effectiveness for subcapsular injection [15]. Cheng et al. [34] reported that subcapsular transplanted MSC survived for longer periods, but they did not enter the renal parenchyme. It appears that transplanted MSC provide protection against AKI via paracrine signaling rather than by replacement of the damaged cells.

Clearly, renal arterial injection can deliver cells efficiently by avoiding filtering organs; thus, it is expected to provide the kidney with the highest cell concentration. Our previous study also showed significant effectiveness of renal arterial infusion of SVF [15]. However, our present study did not replicate this finding. In our present study, the renal arterial injection was performed via aortic puncture, not direct renal arterial puncture, which was used in our previous study. This might be one of the possible explanations for the differences between our two studies.

Moreover, intracoronary arterial injection of cultured cells has been reported to cause microvascular obstruction due to culture hypertrophy [35, 36]. Tatsumi et al. [37] showed that tissue factor (TF) expression on the cell surface was responsible for promoting ADSC-mediated blood clotting. Unlike ADSC, SVF showed little coagulant activity in an *in vitro* coagulation assay. TF is rarely detected on the cell surface of SVF by immunostaining, and TF mRNA expression in SVF is only a tenth of that in ADSC. Since renal arterial injection of cultured cells can result in vascular obstruction, renal parenchymal injection can achieve maximum effectiveness while using a limited number of cells and avoiding these possible adverse effects.

Our study has several limitations. First of all, the exact composition of SVF has not been confirmed. The secretion of SVF and ADSC was also not investigated. This is an important process to verify the hypothesis that renal function was protected by paracrine effect without engraftment. Second, the rats were followed for only 2 weeks. Longer studies are needed to evaluate the effectiveness of cell-based therapies for ischemic AKI and to determine whether they can prevent progression to chronic kidney disease. Zhou et al. [38] reported that preischemic administration of SVF reduced the serum creatinine level six months after ischemic AKI and inhibited renal fibrosis, slowing the progression of chronic kidney disease. Third, the rats were injected only once with a fixed dose (1 × 10^6^) applied concurrently with ischemia. If repeated injections appear to be synergistic, ADSC are a better choice because they can be stored in the freezer without significantly compromising cell viability and can be easily expanded without requiring additional liposuction. Fourth, there have been reports that female rodents were more resistant to ischemic AKI than male rodents [39]. It is questionable whether the results of male-only study can be equally applied to female. Lastly, the baseline CrCl was different among the groups, interfering with the analysis of the results. In subsequent experiments, the baseline CrCl should be measured on all subjects before randomization, and the subjects should be matched by CrCl to equalize the groups at baseline. Nevertheless, the findings from our study suggest that renal parenchymal injection of SVF holds promise as a simple therapeutic approach for the treatment of predictable injuries that might be induced by kidney surgery, such as nephron-sparing surgery and kidney transplantation from deceased donors.

## 5. Conclusion

Our findings suggest that renal function is effectively protected from ischemic AKI through renal parenchymal injection of SVF, which has enhanced antifibrotic, antiapoptotic, and antioxidative effects. Our findings have provided important clues for conducting future research, including large animal experiments and clinical studies.

## Data Availability

The data used to support the findings of this study are available from the corresponding author upon request.

## Supplementary Materials

Supplementary 1 Figure S1: Renal index. NS, *p* > 0.05; ^∗^*p* < 0.05; ^∗∗∗^*p* < 0.001 compared with the AKI control group.

Supplementary 2 Figure S2: determination of CM-DiI labeled SVF and ADSC. CM-DiI labeled SVF and ADSC were observed by fluorescent microscope. Scale bar 2000 *μ*m.
