# Supplementary figures and images for: Comparison of Stromal Vascular Fraction and Adipose-Derived Stem Cells for Protection of Renal Function in a Rodent Model of Ischemic Acute Kidney Injury
Source: Stem Cells Int. 2022 May 6;2022:1379680. doi: 10.1155/2022/1379680 (PMC9107055; doi:10.1155/2022/1379680)

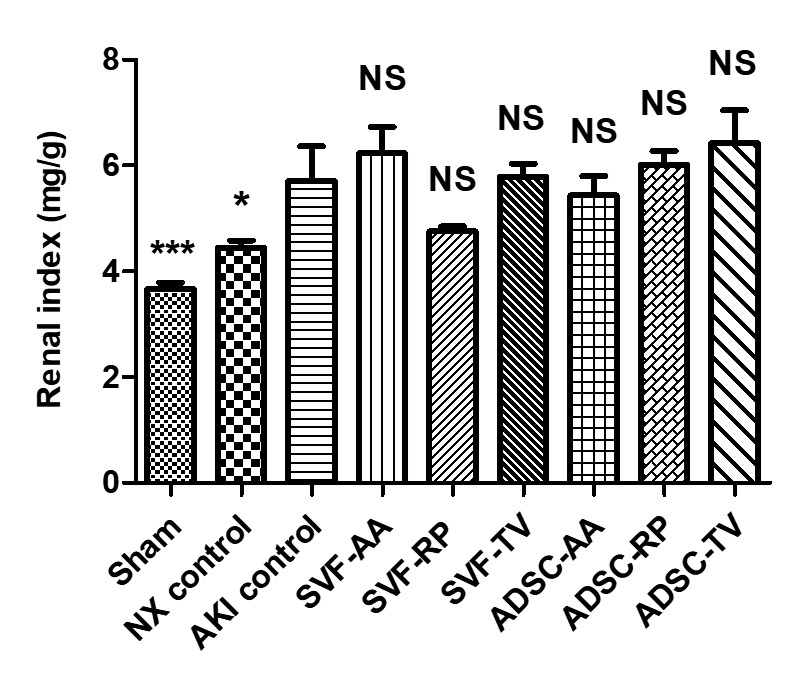

Supplement: Supplementary 1 — Figure S1: Renal index. NS, p > 0.05; ∗p < 0.05; ∗∗∗p < 0.001 compared with the AKI control group. [file 1379680.f1.jpg]

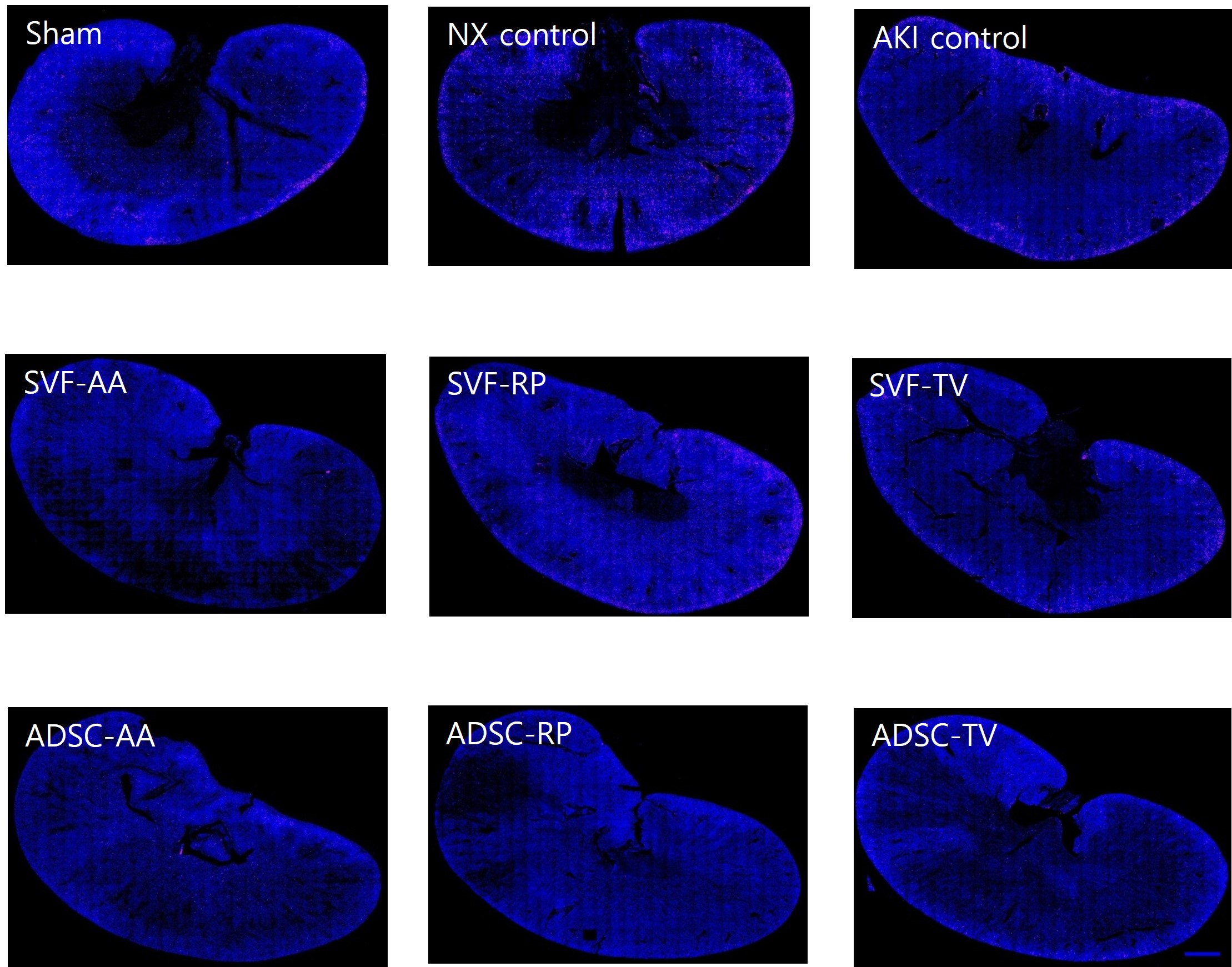

Supplement: Supplementary 2 — Figure S2: determination of CM-DiI labeled SVF and ADSC. CM-DiI labeled SVF and ADSC were observed by fluorescent microscope. Scale bar 2000 μm. [file 1379680.f2.jpg]
